# Beneficial and limiting factors for return to work following anterior cruciate ligament reconstruction: a retrospective cohort study

**DOI:** 10.1007/s00402-016-2594-6

**Published:** 2016-11-21

**Authors:** Judith A. M. Groot, Freerk J. Jonkers, Arthur J. Kievit, P. Paul F. M. Kuijer, Marco J. M. Hoozemans

**Affiliations:** 1 CORAL—Center for Orthopaedic Research Alkmaar, Orthopaedic Department, Noordwest Ziekenhuisgroep, Alkmaar, The Netherlands; 2 Department of Surgery, Onze Lieve Vrouwe Gasthuis, Amsterdam, The Netherlands; 3 Coronel Institute of Occupational Health, Academic Medical Center, University of Amsterdam, Amsterdam, The Netherlands; 4 Faculty of Behavioural and Movement Sciences, Department of Human Movement Sciences, Vrije Universiteit Amsterdam, MOVE Research Institute Amsterdam, Van der Boechorststraat 9, 1081 BT Amsterdam, The Netherlands

**Keywords:** Return to work, Anterior cruciate ligament, Work load, ACL reconstruction

## Abstract

**Purpose:**

Evidence-based advice for return to work (RTW) after anterior cruciate ligament (ACL) reconstruction is not available. Therefore, the objectives of this study were to determine when patients achieve full RTW, and to explore the beneficial and limiting factors for fully RTW after ACL reconstruction.

**Methods:**

A retrospective cohort study was performed after ACL reconstruction among 185 patients in one hospital. Data from patient files and a questionnaire were used to explore whether patient-, injury-, surgery-, sports-, work- and rehabilitation-related factors are beneficial or limiting for fully RTW after ACL reconstruction, using a backward stepwise logistic regression analysis.

**Results:**

Of the 125 (68%) patients that returned the questionnaire, 36 were not part of the working population. Of the remaining 89 patients, 82 patients (92%) had returned fully to work at follow-up. The median time to fully RTW was 78 days. In the final regression model, which explained 29% of the variance, a significant OR of 5.4 (90% CI 2.2–13.1) for RTW > 78 days was observed for patients performing heavy knee-demanding work compared to patients performing light knee-demanding work. In addition, a significant and positive OR (1.6, 90% CI 1.2–1.9) for the number of weeks walking with the aid of crutches for RTW > 78 days was observed in the final model.

**Conclusion:**

After ACL reconstruction, 92% of the patients fully return to work at a median time of 78 days. The significant predictors for fully RTW > 78 days are performing heavy knee-demanding work and a longer period of walking aided with crutches after ACL reconstruction.

## Introduction

A complete or partial tear of the anterior cruciate ligament (ACL) is one of the most common knee injuries [1–3]. Young adults (17–45 years) are at higher risk for ACL injury, especially those who are active in pivoting sports, such as soccer and skiing [4–6]. In most cases, the ACL injury occurs during sports participation and usually follows an abrupt deceleration, i.e., jumping or sideways cutting [7, 8]. In approximately 50–70% of patients, ACL rupture is accompanied by additional meniscal injury, cartilage damage, and/or posterior or collateral ligament ruptures [9, 10].

Although conservative treatment for ACL rupture can be effective, young and active patients are often treated by surgical reconstruction [11]. Patients that are active in pivoting sports are more likely to successfully return to the initial sport when treated by ACL reconstruction as compared with conservative therapy [12, 13]. Furthermore, ACL reconstruction is recommended if patients complain of knee instability in common daily activities and work [14]. Optimal timing for an ACL reconstruction is when the range of knee motion has normalized and the synovial reaction in the knee has diminished [2, 12, 14]. A longer period between ACL injury and ACL reconstruction could increase the risk of meniscal injuries and/or cartilage damage due to prolonged instability [12, 15, 16].

Typically, the goal of ACL reconstruction and the accompanying rehabilitation program is return to sports [14]. Recent literature provides detailed information about the predictive factors for adequate return to sports [2, 11, 17–21]. The ability of patients to return to sports after ACL reconstruction is governed by various factors which include the patient’s characteristics (e.g., gender or age) [11, 19], findings of surgery (e.g., injury grade or joint laxity) [11, 17, 19], knee function before the ACL reconstruction (e.g., muscle strength or flexibility) [2, 17, 19, 20], physical activity level before ACL reconstruction (e.g., practicing sports or Tegner activity level) [2, 17, 20], and psychological factors (e.g., confidence or motivation) [20, 21]. However, besides return to the previous sport levels, ACL reconstructions may be necessary to adequately perform daily activities and return to work (RTW) [14]. If ACL reconstruction is required to perform activities at work, a rapid rehabilitation and RTW have the potential to benefit patients, employers, and society as a whole [22]. This may concern a rather large population as it is estimated that about 40% of the Dutch population in the age range of 15–25 years and about 85% in the age range of 25–45 years are part of the working population. Therefore, the likelihood of a patient with an ACL injury to be working is high. However, as far as we are aware of, there is no literature on beneficial and limiting factors for RTW after ACL reconstruction. Consequently, evidence-based advice for optimal RTW after ACL reconstruction is not available. Therefore, the two objectives of this study were: (1) to determine when patients achieve full RTW after ACL reconstruction; and (2) to explore whether, and to what extent, patient characteristics, injury and surgery-related characteristics, sports and work-related characteristics, and characteristics of rehabilitation are beneficial or limiting factors for fully RTW in patients that have undergone ACL reconstruction.

## Methods

### Study design and population

A retrospective cohort study was combined with a cross-sectional questionnaire survey. All consecutive patients, aged 18–65 years, who underwent primary ACL reconstruction (single bundle semitendinosus-gracilis autograft) at Noordwest Ziekenhuisgroep, Alkmaar, the Netherlands, between April 1st 2012 and April 1st 2014, were screened for inclusion. Patients included had to be part of the working population and have sufficient knowledge of the Dutch language to complete a questionnaire to be eligible for inclusion. Patients were excluded if they had concomitant collateral ligament injuries and/or posterior cruciate injury and/or ACL revision. This study was performed in accordance with the guidelines on Good Clinical Practice of the International Conference on Harmonisation [23] and the Declaration of Helsinki [24]. However, medical ethical approval of the study was not required.

### Procedure

The electronic database of the hospital was searched for patients that fulfilled the inclusion criteria listed above. All patients eligible for inclusion received an invitation letter, between April 1st 2013 and June 1st 2014, containing information regarding the study, an informed consent form, a questionnaire and a stamped return envelope. Patients not responding to this invitation were contacted by telephone and asked if they had received the invitation letter. If not, a new invitation was sent. If so, they were asked to respond to the invitation. Reasons for not wishing to participate were registered if given. The files of patients that agreed to participate were screened and relevant information was extracted.

### Return to work

The primary outcome variable was the number of days between the date of surgery and the date the patient fully returned to work. To identify potential beneficial and limiting factors for a rapid RTW, the continuous RTW variable was dichotomized into rapid and prolonged RTW with the median value of RTW as cut-off point [25, 26].

### Beneficial and limiting factors for RTW

The independent (predictor) variables of interest on the basis of previous literature and also on clinical expertise were patient-, injury-, surgery-, sports-, work-, and rehabilitation-related factors (Table 1). These factors were retrospectively assessed from the patient files or the questionnaire.

**Table 1:** Dependent and independent variables

Variables	Time of measurement	Clarification
Dependent		
RTW (return to work)	By questionnaire at follow-up	RTW was defined as the number of days between the date of surgery and the date the patient (fully) RTW. RTW was dichotomized into ‘rapid RTW’ and ‘prolonged RTW’ using the median of the number of days between surgery and (full) RTW as cut-off point
Independent: patient characteristics		
Gender	Extracted from patient files	Male–female
Age	Extracted from patient files	Age at time of surgery in years
BMI	Extracted from patient files	Weight/Length^2^, BMI was dichotomized into ≤24 and > 24 using the median of the BMI
IKDC	By questionnaire at follow-up	The IKDC contains 18 items that measure symptoms, function, and sports activities in patients with a variety of knee problems. The score can vary between 0 and 100. A higher score means a higher level of functioning and a lower level of symptoms
EQ-5D	By questionnaire at follow-up	The questionnaire measures five dimensions: mobility, self-care, daily activities, pain/discomfort and anxiety/depression, and a visual analog scale for overall health. Each dimension can be assessed at three levels: no problems (1), some problems (2), and extreme problems (3). These health profiles can be converted into a weighted health index for each patient. Dutch tariffs were used to estimate the weighted health index. The weighted health index gives a number between 0 (death) and 1 (complete healthy)
Independent: injury-related characteristics		
Side	Extracted from patient files	Right-left
Knee extension	Extracted from patient files	Dichotomized into ‘full knee extension’ and ‘no full knee extension’
Time injury to surgery	Extracted from patient files	Time between injury and surgery, dichotomized into ≤3 months and > 3 months
Independent: characteristics of surgery		
Medial menisectomy	Extracted from patient files	Menisectomy done by surgeon. Yes–No
Lateral menisectomy	Extracted from patient files	Menisectomy done by surgeon. Yes–No
Cartilage damage	Extracted from patient files	Assessed by surgeon using the guidelines of the International Cartilage Repair Society, grade zero (normal) up to grade four (severe abnormal)
Independent: sports activity level		
Knee-taxing sport	Extracted from patient files	Practicing knee-taxing sports before ACL injury? Yes–No
Contact sports	Extracted from patient files	Practicing contact sports before ACL injury? Yes–No
Pivoting sports	Extracted from patient files	Practicing pivoting sports before ACL injury? Yes–No
Tegner score before injury	Extracted from patient files	The patients had to rate their level of activity with an integer between 0 (‘sick leave or disability pension because of knee problems’) and 10 (‘competitive sports, soccer—national and international elite’)
Tegner score after injury before surgery	Extracted from patient files	The patients had to rate their level of activity with an integer between 0 (‘sick leave or disability pension because of knee problems’) and 10 (‘competitive sports, soccer—national and international elite’)
Contact during injury	By questionnaire at follow-up	Was there physical contact during the ACL injury? Yes–No
Independent: work-related characteristics		
Knee-demanding work	By questionnaire at follow-up	Knee-demanding work is measured with the WORQ. Knee-demanding work is defined as often or nearly always performing crouching, kneeling, clambering, lifting or carrying or taking the stairs during work
WORQ experience	By questionnaire at follow-up	The trouble the patient experiences during work measured with the WORQ. It generates a score between 0 and 100. A lower score indicates that the patient experiences more trouble during knee-related activities
Independent: characteristics of rehabilitation		
Physiotherapy up to 6 weeks (frequency per week)	By questionnaire at follow-up	Frequency of physiotherapy, dichotomized into ≤2 times a week and >2 times a week
Physiotherapy 6 weeks up to 3 months (frequency per week)	By questionnaire at follow-up	Frequency of physiotherapy, dichotomized into ≤2 times a week and >2 times a week
Gym	By questionnaire at follow-up	Perform exercises in the gym. Yes–no
Homework exercise	By questionnaire at follow-up	Perform homework exercises. Yes–no
Crutch walking	By questionnaire at follow-up	Number of weeks crutch walking

### Patient factors

Patient characteristics assessed from the patient files were gender (male–female), date of birth, and body mass index (BMI). Pain, knee disability, and function were assessed using the validated Dutch version of the subjective International Knee Documentation Committee (IKDC) [27, 28]. The IKDC contains 18 items that measure symptoms, function, and sports activities in patients with a variety of knee problems. The IKDC was proven valid when evaluating patients with recent ACL ruptures or those within 1 year of an ACL reconstruction [27, 28]. The score varies between 0 and 100. A higher score means a higher level of function and a lower level of symptoms. In addition, the quality of life (QoL) as perceived by the patient was assessed using the EuroQol-5D (EQ-5D). This questionnaire is a generic standardized health-related QoL measurement tool and is widely used in clinical trials, observational studies, and other studies. The questionnaire measures five dimensions: mobility, self-care, daily activities, pain/discomfort, and anxiety/depression. Each dimension can be assessed at three levels: no problems, some problems, and extreme problems. These five dimensions provide a health profile that can be converted into a weighted health index for each patient. In this study, Dutch tariffs were used to estimate the weighted health index [29]. The weighted health index results in a score between 0 (death) and 1 (completely healthy). The EQ-5D uses also a visual analog scale for overall health.

### Injury-related factors

Injury-related characteristics assessed from the patient files were the side of the ACL injury (left–right), extension lag of the affected knee before the ACL surgery (in degrees), and time interval between injury and surgery (dichotomized into short or prolonged with a cut-off point at three months [14, 15, 30]).

### Surgery-related factors

Characteristics of surgery assessed from the patient files were meniscal injury as observed by the surgeon (yes–no) and cartilage damage assessed using the guidelines of the International Cartilage Repair Society, grade zero (normal) up to grade four (severely abnormal) [31].

### Sports-related factors

Sports characteristics assessed using the questionnaire were practicing knee-demanding sports before injury (yes–no), practicing contact sports before injury (yes–no), and practicing pivoting sports before injury (yes–no). In addition, the activity level was assessed using the Tegner Activity Score (TAS). The TAS is retrieved from patient files before ACL injury and between ACL injury and ACL surgery, both assessed one week before surgery by the physician assistant. At follow-up, the TAS was assessed by means of a questionnaire. For this, patients had to rate their level of activity by an integer between 0 (‘sick leave or disability pension because of knee problems’) and 10 (‘competitive sports, soccer—national and international elite’) [32, 33]. In addition, the patients were asked whether they had physical contact with another person at the time of injury (yes–no).

### Work-related factors

Work-related characteristics were assessed using the Work Osteoarthritis or joint Replacement Questionnaire (WORQ) to determine knee-demanding workload [34]. This is a questionnaire for RTW, first validated for patients with a Total Knee Prosthesis [22]. The questionnaire focuses on knee-demanding activities during work. Heavy knee-demanding work in this study is defined as ‘often’ or ‘nearly always’ performing one of the following five work-related activities: crouching, kneeling, clambering, lifting or carrying, or taking the stairs [22]. It also assesses the difficulty patients experience when performing the above-mentioned set of knee-demanding activities during work. A score between 0 and 100 is generated, a lower score indicating that the patient experiences more difficulty during knee-demanding activities.

### Rehabilitation-related factors

Characteristics of rehabilitation assessed using the questionnaire were the number of physiotherapy visits per week and the duration of physiotherapy after surgery in months, additional fitness training during rehabilitation (yes–no), additional home exercise performed during rehabilitation (yes–no), and number of weeks of walking with the aid of crutches.

### Statistical analyses

Descriptive statistics were used to characterize the study population. Mean and standard deviation (*SD*) were used for normally distributed variables, and median and interquartile range (*IQR*) for not normally distributed variables. The normality of distributions of the variables was explored visually using histograms, q–q plots, and box-plots, and using the Kolmogorov–Smirnov test.

To determine whether the respondents of the questionnaire were representative for the study population, a non-response analysis was performed. Patient and surgical characteristics from the patient files were compared between the patients that responded to the questionnaire and those that did not. In the case of normally distributed continuous variables, independent *t* tests were performed. In the case of not normally distributed continuous variables, the Mann–Whitney *U* test was used and for categorical or dichotomous variables differences were explored using Chi-square tests.

The number of days between the date of surgery and the date the patient fully returned to work was used to determine when patients achieved full RTW. The continuous RTW variable was dichotomized into rapid and prolonged RTW with the median value of RTW as cut-off point [25, 26]. To explore whether and which of the independent variables were most strongly associated with the dependent variable fully RTW, a logistic backward stepwise regression was used. Hot deck imputation was used for missing data [35]. To further reduce the number of independent variables that were initially entered into the regression model, considering the number of cases in the analyses, only those independent variables were used that were univariately associated with fully RTW at a *p* value of ≤0.25 [36]. Before running the logistic backward stepwise regression analyses, the independent variables were checked for multicollinearity (*r* > 0.80) [37]. For the remaining independent variables, the logistic backward stepwise regression was performed with time between surgery and filling in the questionnaire forced into the regression. For each of the remaining independent variables that remained in the regression model after backward stepwise regression, their odds ratios (OR) and corresponding 90% confidence intervals (CI) were determined. Because this is the first study on this topic, a 90% CI was chosen to prevent possibly relevant clinical variables being opted out. The overall fit of the logistic regression models was quantified by Nagelkerke’s *R* ^2^ (*R*_N_^2^), which can be interpreted as *R* ^2^ in linear regression [38].

All statistical analyses were performed using IBM SPSS Statistics version 20.0 (IBM Corporation, Armon, NY, USA).

## Results

### Participants

**Fig. 1:**
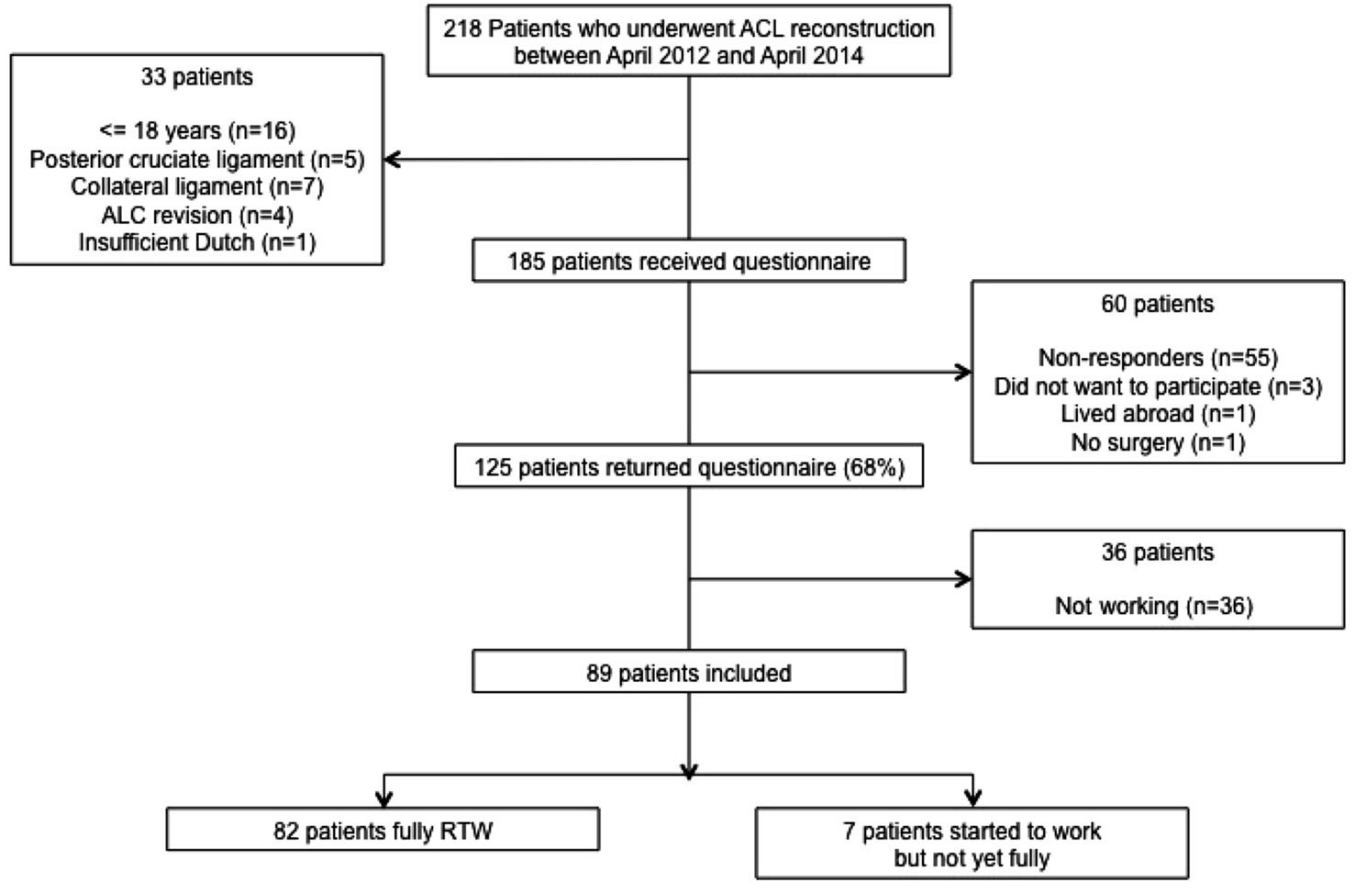
Flow diagram of patients’ participation. *ACL* anterior cruciate ligament

Patient inclusion is displayed in a flow diagram (Fig. 1). A total of 218 patients had received an ACL reconstruction in the hospital in the period between April 1st 2012 and April 1st 2014. After the initial screening, 33 patients appeared not to be eligible for inclusion, because they were younger than 18, had other ligament injuries, had an ACL revision, or had insufficient knowledge of the Dutch language. Therefore, 185 patients were contacted and received a questionnaire. One hundred and twenty-five (125) patients returned the questionnaire, a response rate of 68%. Only five patients gave a reason for not returning the questionnaire: they did not want to participate, lived abroad, or had no surgery. Additional 55 patients did not respond at all. Non-response analysis showed no significant differences in pre-operative age, body mass index, TAS before ACL injury, TAS between ACL injury and ACL reconstruction, meniscal injury, or cartilage damage between the response and non-response group.

### Descriptives

Descriptive data of the study participants are displayed in Table 2. Study participants had a median (*IQR*) age at surgery of 31 (24–44) years, a mean (SD) body mass index of 24 (3) kg/m^2^, a median (*IQR*) TAS before ACL injury of 7 (6–9), a mean (*SD*) TAS between ACL injury and surgery of 3 (1), and a median (*IQR*) time from injury to surgery of 8 (4–13) months. Characteristics of study participants (median (*IQR*)) assessed by follow-up questionnaire were an IKDC score of 81 (66–89), an EQ-5D index score of 0.8 (0.8–1), a WORQ difficulty score of 89 (77–95), and a number of weeks of crutch walking of 5 (3–6) weeks.

**Table 2:** Characteristics of study participants (*N* = 82)

Patient characteristics	Categories	All, *N* = 82	RTW > 79 days (*N* = 41)	RTW ≤ 78 days (*N* = 41)	Missing
Gender (*n*, %)	Male	43 (52)	21 (51)	22 (54)	0
	Female	39 (48)	20 (49)	19 (46)	
Age (*n*, %)	>27	52 (63)	27 (66)	25 (61)	0
	≤27	30 (37)	14 (34)	16 (39)	
BMI (*n*, %)	>24	42 (51)	21 (51)	21 (51)	0
	≤24	40 (49)	20 (49)	20 (49)	
IKDC^a^	–	81 (65–88)	64 (54–76)	78 (64–87)	14
EQ-5D^a^	–	0.8 (0.8–1)	0.8 (0.8–1)	1 (0.8–1)	0
Injury-related characteristics					
Side (*n*, %)	Left	37 (45)	21 (51)	16 (39)	0
	Right	45 (55)	20 (49)	25 (61)	
Knee extension (*n*, %)	Not full	3 (4)	3 (7)	0 (0)	3
	Full	76 (96)	38 (93)	38 (100)	
Time injury to surgery (*n*, %)	>3 months	70 (85)	37 (90)	33 (80)	0
	≤3 months	12 (15)	4 (10)	8 (20)	
Tegner score before injury^a^	–	8.5 (7–9)	7 (5.5–9)	7 (7–9)	35
Tegner score after injury before surgery^a^	–	4 (3–4)	3 (2–4)	3 (2–4)	34
Contact during trauma (*n*, %)	Physical contact	19 (23)	11 (27)	8 (19)	0
	No contact	63 (77)	30 (73)	33 (81)	
Characteristics of surgery					
Medial menisectomy (*n*, %)	Menisectomy	13 (17)	7 (17)	6 (17)	6
	Non-menisectomy	63 (83)	33 (83)	30 (83)	
Lateral menisectomy (*n*, %)	Menisectomy	9 (12)	4 (10)	5 (14)	6
	Non-menisectomy	67 (88)	36 (90)	31 (86)	
Cartilage damage (*n*, %)	Grade 3,4	10 (13)	6 (15)	4 (11)	5
	Grade 0, 1, 2	67 (87)	34 (85)	33 (89)	
Sports activity level					
Knee-taxing sport pre trauma (*n*, %)	Knee-taxing	65 (79)	32 (78)	33 (80)	0
	Not knee-taxing	17 (21)	9 (22)	8 (20)	
Contact sport pre trauma (*n*, %)	Contact sports	49 (60)	23 (56)	26 (63)	0
	Non-contact sports	33 (40)	18 (44)	15 (37)	
Pivoting sport pre trauma (*n*, %)	Pivoting	62 (76)	29 (71)	33 (80)	0
	Non-pivoting	20 (24)	12 (29)	8 (20)	
Tegner score before injury^a^	–	8.5 (7–9)	7 (5.5–9)	7 (7–9)	35
Tegner score after injury before surgery^a^	–	4 (3-4)	3 (2–4)	3 (2–4)	34
Contact during trauma (*n*, %)	Physical contact	19 (23)	11 (27)	8 (19)	0
	No contact	63 (77)	30 (73)	33 (81)	
Work-related characteristics					
Workload (*n*, %)	Heavy	38 (46)	26 (63)	12 (29)	0
	Light	44 (54)	15 (37)	29 (71)	
WORQ experience^a^	–	88 (77–95)	90 (79–96)	88 (77–94)	3
Characteristics of rehabilitation					
Physiotherapy up to 6 weeks (*n*, %)	≤2	66 (80)	33 (80)	33 (80)	0
	>2	16 (20)	8 (20)	8 (20)	
Physiotherapy 6 weeks up to 3 months (*n*, %)	≤2	65 (82)	33 (80)	32 (84)	3
	>2	14 (18)	8 (20)	6 (16)	
Gym (*n*, %)	No gym	44 (54)	22 (54)	22 (55)	1
	Gym	39 (46)	19 (46)	18 (45)	
Homework exercises (*n*, %)	No homework exercise	7 (8)	3 (7)	4 (10)	0
	Homework exercise	75 (92)	38 (93)	37 (90)	
Weeks using crutches^a^	–	4 (2–6)	5 (4–7)	4 (3–6)	3

^a^Median (interquartile range)

### RTW

Of the 125 patients that received the questionnaire, 36 patients had no work and were not part of the working population, and so they were excluded. Thus, 89 patients were eligible for inclusion. Eighty-two patients (92%) had fully returned to work at the moment of filling in the questionnaire, and seven patients had started to work but had not yet returned to their full work activities (Table 2). The median fully RTW time was 78 days with an IQR of 49–112 days.

### Beneficial and limiting factors for RTW

Three predictor factors were associated with the binary outcome variable fully RTW (≤78, >78 days) at a *p* value equal to or less than 0.25 in the univariate analyses (Table 3). Time between ACL injury and ACL reconstruction (*p* = 0.22), knee-demanding work (*p* ≤ 0.01), and number of weeks walking with the aid of crutches (*p* = 0.01) were entered in the backward stepwise regression analysis with time between surgery and filling in the questionnaire forced in the model. Table 4 presents the final model. The odds for fully RTW ≤78 days were 5.4 times greater for patients who perform light knee-demanding work compared with patients who perform heavy knee-demanding work (OR = 5.40, 90% CI 2.24–13.06). Furthermore, also walking aided by crutches was significantly associated with fully RTW with the OR (OR = 1.47, 90% CI 1.16–1.85), indicating an increase in risk for RTW > 78 days for an increase in the number of weeks that patients needed to walk with crutches. The model explained 29% of the variance of fully RTW (Nagelkerke *R* ^2^).

**Table 3:** Results of the univariate associations between the predictor variables and fully RTW > 78 days using logistic regression analyses

Patient characteristics	Categories	OR	90% CI for OR		*p* value
			Lower	Upper	
Gender	Male	0.91	0.44	1.88	0.83
	Female (ref)				
Age	>27	1.23	0.58	2.63	0.65
	≤27 (ref)				
BMI	>24	1.00	0.48	2.07	1.00
	≤24 (ref)				
IKDC*	–	1.01	0.99	1.04	0.41
EQ-5D*	–	0.25	0.02	3.41	0.38
Injury-related characteristics					
Side	Left	1.64	0.79	3.42	0.27
	Right (ref)				
Knee extension	Not full	1.01	0.99	1.02	0.49
	Full (ref)				
Time injury to surgery	>3 months	2.24	0.76	6.61	**0.22**
	≤ 3 months (ref)				
Characteristics of surgery					
Medial menisectomy	Menisectomy	1.06	0.39	2.90	0.92
	Non-menisectomy (ref)				
Lateral menisectomy	Menisectomy	0.69	0.21	2.23	0.60
	Non-menisectomy (ref)				
Cartilage damage	Grade 3,4	1.46	0.47	4.53	0.59
	Grade 0,1,2 (ref)				
Sports activity level					
Knee-taxing sport pre trauma	Knee-taxing	0.86	0.35	2.12	0.79
	Not knee-taxing (ref)				
Contact sport pre trauma	Contact sports	0.74	0.35	1.55	0.50
	Non-contact sports (ref)				
Pivoting sport pre trauma	Pivoting	0.59	0.25	1.38	0.31
	Non-pivoting (ref)				
Tegner score before injury*	–	0.87	0.69	1.10	0.33
Tegner score after injury before surgery*	–	0.83	0.58	1.17	0.37
Contact during trauma	Physical contact	1.51	0.63	3.61	0.43
	No contact (ref)				
Work-related characteristics					
Workload	Heavy	4.19	1.93	9.11	**<0.01**
	Light (ref)				
WORQ experience*	–	1.00	0.98	1.03	0.87
Characteristics of rehabilitation					
Physiotherapy up to 6 weeks (frequency per week)	≤2	1.00	0.40	2.50	1.00
	>2 (ref)				
Physiotherapy 6 weeks up to 3 months (frequency per week)	≤2	0.77	0.29	2.06	0.67
	>2 (ref)				
Gym	No gym	0.95	0.46	1.97	0.90
	Gym (ref)				
Homework exercises	No homework exercise	0.73	0.20	2.71	0.69
	Homework exercise (ref)				
Weeks crutches*	–	1.37	1.12	1.67	**0.01**

*Ref* reference category

**Table 4:** Final regression model

Variables	OR	90% CI for OR	
		Lower	Upper
Time between surgery and filling in the follow-up questionnaire	1.01	0.99	1.02
Workload			
Heavy knee-demanding	5.40	2.24	13.06
Light knee-demanding	1.00	–	–
Use of crutches (weeks)	1.57	1.17	1.85

Nagelkerke *R* square 0.285

## Discussion

This is the first study to be conducted describing RTW after ACL reconstruction. Following ACL reconstruction, 92% of the patients fully returned to work with a median time interval of 78 days. Heavy knee-demanding work and more weeks in which walking was aided by crutches are limiting factors for rapid RTW after ACL reconstruction. As far as we know, no other study has described the impact of ACL reconstruction on RTW, making a direct comparison with other studies difficult.

### Beneficial and limiting factors for RTW

The strongest predictive factor for RTW after ACL reconstruction was the level of knee-demanding work, assessed with the WORQ [34]. One study [22] previously used the WORQ for defining the level of knee-demanding work and a similar definition is also used in the current study. The WORQ was applied in patients that received a primary total knee arthroplasty. When comparing the time to fully RTW after a total knee arthroplasty with the time to fully RTW after ACL reconstruction, the results are better for ACL reconstruction, with 71% of ACL patients returning within 3 months, while 50% of total knee arthroplasty patients returned within three months. The rate of RTW is also better for ACL reconstruction compared with total knee arthroplasty, respectively, 92 and 80%. There are no other knee-related surgery studies that describe RTW. Following ACL reconstruction, the first 3 months is most vulnerable for the fixation of the graft. Therefore, it was expected that patients who have knee-demanding work will have a prolonged RTW, because the graft fixation is too weak for knee-demanding work. Thirty-eight patients (46%) performed heavy knee-demanding work in this study. Two-thirds of the patients (68%) that perform heavy knee-demanding work had a prolonged RTW, while one-third of the patients that perform light knee-demanding work had a prolonged RTW.

The other predictive factor that was significantly associated with full RTW > 78 days in the final regression model was the number of weeks that patients needed to walk with the aid of crutches following surgery. The significant OR of 1.54 indicates that for each week, the patient walks longer with the aid of crutches, the risk for prolonged RTW is 1.54 times greater. This means that for each four weeks, the OR increases to 4.57. The number of weeks of walking with the aid of crutches that are needed following ACL reconstruction has never been investigated in the previous studies, as far as we know. The main question is whether walking with the aid of crutches for more weeks—for instance, because the medical specialists advise doing so—actually increases the risk of prolonged RTW. Another reason might be that patients avoid the workplace anyway, because they are walking with the aid of crutches, possibly because they cannot travel to work or they can perform only sedentary work for the first few weeks, instead of their actual knee-demanding tasks, or it was too painful to work. It may be that patients with insufficient knee function need to walk with crutches for a longer period of time and that post-operative knee function is the actual cause of the prolonged RTW. In clinical settings, the patients are usually advised to walk with crutches as long as they have no active knee extension during walking. This suggests that walking with crutches is not an independent predictor for RTW but an intermediate factor for functional recovery. Considering the arguments stated above, both the knee-demanding workload and the number of weeks walking with the aid of crutches should be carefully considered as predictive factors for RTW. For further research, it is recommended to measure post-operative knee function and active knee extension during rehabilitation. In addition, it is recommended to know the primary factors why patients do not return to work in the first few weeks, or understand the factors that ensured RTW. Furthermore, it should be known whether the patient is able to start working, while they are still walking with the aid of crutches. Therefore, more scientific evidence is required for RTW after knee surgery, especially after ACL reconstruction.

### Return to sports

In contrast with the beneficial and limiting factors for return to sports following ALC reconstruction, no significant association was found for factors, such as body mass index, TAS before ACL injury, TAS between ACL injury and ACL reconstruction, physical contact during injury, physiotherapy, additional home exercises, or the other included potential predictive factors (Table 1) [2, 11, 17–21]. Return to sports is possible after 9–12 months of rehabilitation [14], while fully RTW is possible around 3 months after surgery. This difference in time between return to sports and fully RTW might explain the differences in the limiting and beneficial factors between return to sports and RTW. After 9–12 months, the patients will have recovered more than after 3 months: this might explain the difference in factors at stake. Extension lag of the affected knee before the ACL surgery is a limiting factor for return to sports [2, 12, 14]. In this study, there were three patients who had an extension lag before surgery. Since these three patients had a longer fully RTW time than 78 days, it is possible that this factor also has an effect on fully RTW. However, the number of patients is too small to find an effect on RTW. Furthermore, the median age in this study population was 31 years, which is higher than in other ACL studies focusing on return to sports. This can be explained by the inclusion criteria—patients had to be part of the working population. However, with respect to the other characteristics, the study population in this study was comparable with study populations of other ACL studies, so that these cannot account for the differences in beneficial and limiting factors.

### Clinical relevance

This study shows that patients who perform heavy knee-demanding work and need to undergo ACL reconstruction should be advised to discuss with their employers and/or occupational physician the possibility to perform adapted work activities with low demands on the knees. This might result in a more rapid RTW. In addition, with respect to the number of weeks in which walking is aided with crutches, it may be important to practice walking without the aid of crutches and performing active knee extensions guided by a physiotherapist to reduce the number of weeks walking with crutches.

### Strengths and limitations

This was the first, exploratory, study conducted to investigate beneficial and limiting factors for RTW following ACL reconstruction. Both the medical literature concerning the predictive factors for return to sports and clinical expertise were used to determine potential beneficial and limiting factors evaluated in this retrospective cohort study, which resulted in a large number of potential predictive factors. Another strength of the study was the use of several questionnaires with sufficient clinimetric qualities, such as the IKDC, WORQ, and EQ-5D. In addition, since non-response analysis showed no significant differences between the patients that responded (68%) and the group that did not respond, the responders were deemed a representative sample of the actual population. The seven patients that had not yet returned to work did vary largely in the time between ACL reconstruction and filling out the follow-up questionnaire, which means that there was no association between the rate of RTW and follow-up time, this could be expected, since a longer follow-up time increases the chance of fully RTW. Moreover, almost 85% of the patients with a follow-up time less than 3 months returned to work. These results were similar for patients with a follow-up period of up to 24 months, showing that, although time between surgery and follow-up was purposely included in the final regression model, follow-up time could not have biased the results.

An important limitation of the study is that the patients answered the questionnaire retrospectively, thereby introducing the risk of recall bias. We have partly tried to overcome this bias by making categories for number of variables, most often in two categories. A prospective study including the factors found for RTW might give more reliable and precise estimates. Another possible limitation is that several studies have reported that a higher activity level before ACL injury increases the likelihood of return to sports [12, 13]. In this study, the activity level was measured using the TAS. This score has not yet been validated in Dutch, which is potentially a restriction in this study [14]. Moreover, the TAS before ACL injury had a large number of missing values. These missing values were imputed, but might result in imputation bias. This is another argument for conducting a prospective cohort study on RTW and ACL reconstruction.

## Conclusion

Following ACL reconstruction, 92% of the patients in the study population returned to work within 7 months and 50% of those patients returned fully to work within 78 days. Patients that perform heavy knee-demanding work have a higher chance of a prolonged RTW, and the longer patients walk with crutches, the higher the chance of a prolonged RTW.
